# A novel anti-GD2/4-1BB chimeric antigen receptor triggers neuroblastoma cell killing

**DOI:** 10.18632/oncotarget.4670

**Published:** 2015-07-20

**Authors:** Malvina Prapa, Sara Caldrer, Carlotta Spano, Marco Bestagno, Giulia Golinelli, Giulia Grisendi, Tiziana Petrachi, Pierfranco Conte, Edwin M. Horwitz, Dario Campana, Paolo Paolucci, Massimo Dominici

**Affiliations:** ^1^ Department of Medical and Surgical Sciences for Children & Adults, Division of Oncology, University-Hospital of Modena and Reggio Emilia, Modena, Italy; ^2^ Department of Pathology and Diagnostics, University of Verona, Verona, Italy; ^3^ International Centre for Genetic Engineering and Biotechnology, Trieste, Italy; ^4^ Istituto Oncologico Veneto, Padova, Italy; ^5^ Departments of Pediatrics and Medicine, Division of Hematology/Oncology/BMT, Nationwide Children's Hospital, The Ohio State University College of Medicine, Columbus, Ohio, USA; ^6^ Department of Pediatrics, National University of Singapore, Singapore

**Keywords:** GD2, chimeric antigen receptor, anti-GD2 IgM-derived, neuroblastoma, T lymphocytes

## Abstract

Chimeric antigen receptor (CAR)-expressing T cells are a promising therapeutic option for patients with cancer. We developed a new CAR directed against the disialoganglioside GD2, a surface molecule expressed in neuroblastoma and in other neuroectoderm-derived neoplasms. The anti-GD2 single-chain variable fragment (scFv) derived from a murine antibody of IgM class was linked, via a human CD8α hinge-transmembrane domain, to the signaling domains of the costimulatory molecules 4-1BB (CD137) and CD3-ζ. The receptor was expressed in T lymphocytes by retroviral transduction and anti-tumor activities were assessed by targeting GD2-positive neuroblastoma cells using *in vitro* cytotoxicity assays and a xenograft model. Transduced T cells expressed high levels of anti-GD2 CAR and exerted a robust and specific anti-tumor activity in 4- and 48-hour cultures with neuroblastoma cells. Cytotoxicity was associated with the release of pro-apoptotic molecules such as TRAIL and IFN-γ. These results were confirmed in a xenograft model, where anti-GD2 CAR T cells infiltrating tumors and persisting into blood circulation induced massive apoptosis of neuroblastoma cells and completely abrogated tumor growth. This anti-GD2 CAR represents a powerful new tool to redirect T cells against GD2. The preclinical results of this study warrant clinical testing of this approach in neuroblastoma and other GD2-positive malignancies.

## INTRODUCTION

Neuroblastoma is the most common extra-cranial solid tumor in young children, appearing at diagnosis in more than half of patients as metastatic disease [1]. Survival rates for patients with low and intermediate-risk neuroblastoma have progressively improved but the prognosis for those disseminated or high-risk diseases remains poor [2].

GD2 is a disialoganglioside highly expressed in several pediatric and adult cancers, including neuroblastoma [3, 4]. GD2 is expressed during fetal development but, among normal post-natal tissues, its expression is limited with low levels of expression on osteoprogenitors, brain, peripheral nerves and skin melanocytes [5, 6]. Because of its high surface expression on tumor cells and low expression on normal tissues, GD2 has been a target for the development of immunotherapeutic monoclonal antibodies [7]. Starting from these encouraging clinical results, anti-GD2 antibody therapy is included in many frontline protocols for neuroblastoma [3, 8]. An alternative strategy to antibody therapy relies in incorporating the antibody specificity into a chimeric antigen receptor (CAR) and use this CAR to redirect T cells [9, 10]. In recent clinical trials, CAR T cells directed against CD19 have produced dramatic clinical responses in patients with B cell malignancies [11].

We generated a novel anti-GD2 CAR consisting of a mouse IgM derived anti-GD2 single-chain variable fragment (scFv) linked through the human CD8α hinge-transmembrane domain to a human portion of the 4-1BB costimulatory molecule fused with the human CD3-ζ chain signaling domain. T cells carrying the anti-GD2 CAR were then tested against neuroblastoma cells *in vitro* and *in vivo* xenograft studies.

## RESULTS

### GD2 CAR retroviral vector retains significant transduction efficiency in T cells

**Figure 1:**
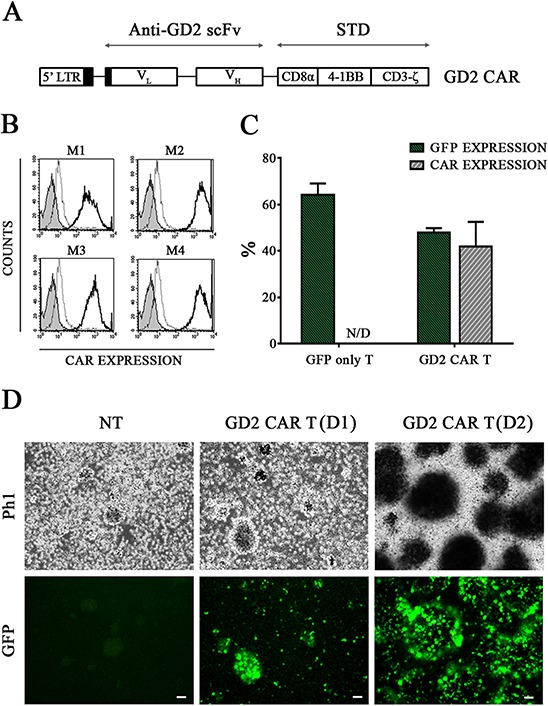
T cells are effectively transduced with GD2 CAR encoding vector **A.** The GD2 CAR construct. The IgM derived anti-GD2 scFv is linked to the signal transduction domain (STD). **B.** Replicate samples of anti-GD2 immunized mice sera (M1, M2, M3 and M4) efficiently recognize GD2 CAR on FLYRD18 cell surface and are introduced for GD2 CAR detection on transduced T cells. Isotype (gray), APC-secondary Ab (broken/gray line) and GD2 positivity (black line). **C.** GD2 CAR T cells were analysed for both GFP and CAR expression levels (48 ± 2% and 40 ± 10%, respectively, *p* > 0.05 by *t*-test) while GFP only T cells were exclusively GFP positive (64 ± 5%) Mean ± SEM. **D.** representative phase 1 (Ph1) and green fluorescence (GFP) photomicrographs (scale bar, 50 μm) of non-transduced (NT) pre-stimulated T cells (left panel) and GD2 CAR T cells after gene transfer revealing GFP-positive clusters in two representative donors (D) in middle (D1) and right (D2) panels.

The ectodomain of the CAR used in this study was a single-chain variable fragment (scFv) derived from a mouse IgM anti-GD2 MoAb in which heavy (V_H_) and light (V_L_) variable fragments were joined by 18 amino acids encoding the linker sequence, allowing the correct folding of the antigen binding site [12]. The scFv sequence was fused with the human CD8α derived hinge-transmembrane domain that connects to a signal transduction domain, consisting of 4-1BB and CD3-ζ (Fig. 1A). This CAR was expressed by a retroviral vector into human T cells.

To determine GD2 CAR expression in T cells, we generated anti-idiotypic antibodies specific for the anti-GD2 scFv. Immunized animal sera were obtained and titrated by flow cytometry on transduced FLYRD18 cells known to retain high levels of transgene expression by GFP analyses. All obtained sera efficiently recognized GD2 CAR (Fig. 1B) on FLYRD18 surface and were therefore applied to detect GD2 CAR in the study and, as shown in Fig. 1C, GD2 CAR was significantly expressed after retroviral transduction on T cells.

**Figure 2:**
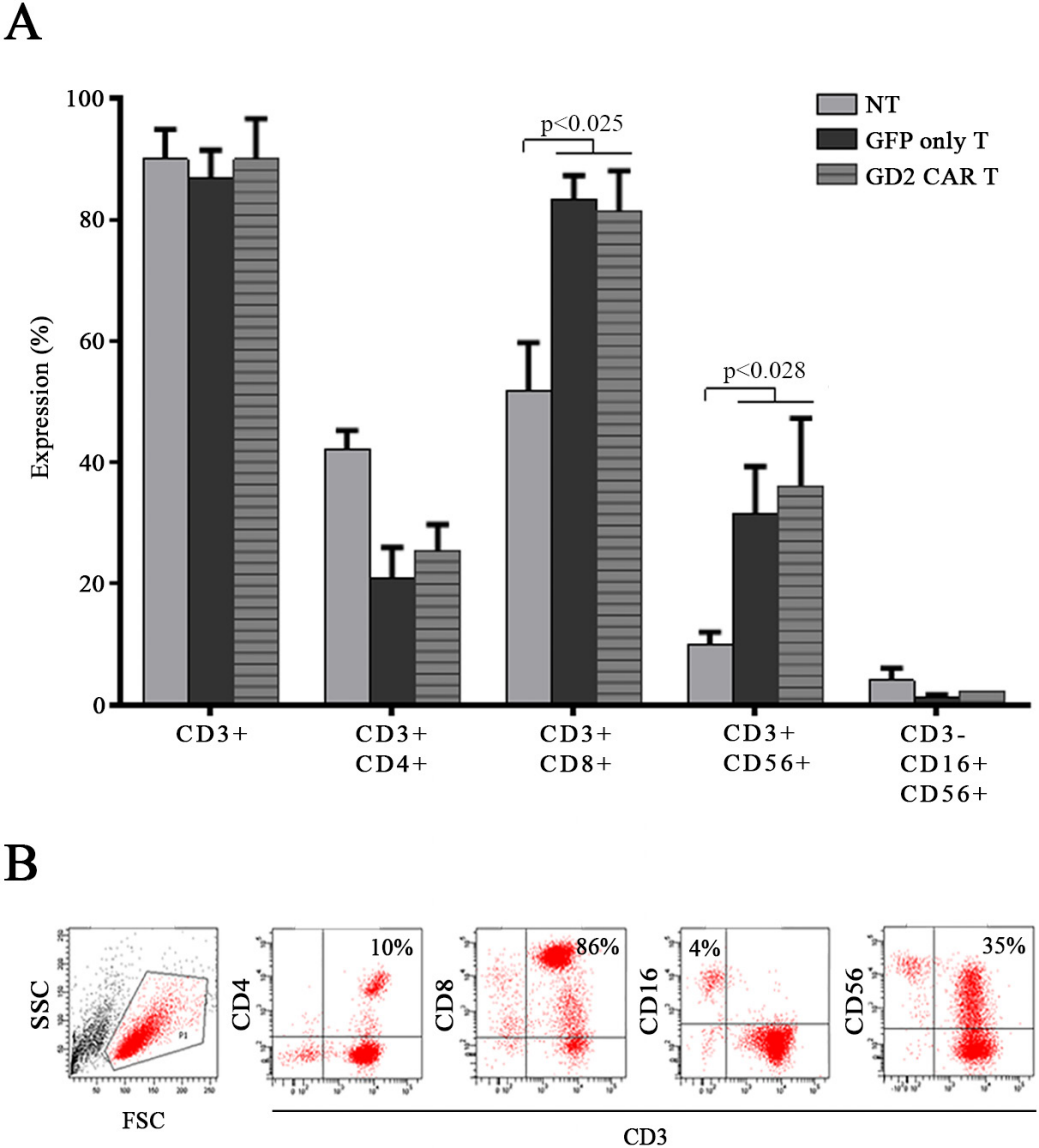
Effectors characterization **A.** non-transduced T cells (NT), GFP only T cells and GD2 CAR T cell sub-populations assessed by flow cytometry: both GFP only T cells and GD2 CAR T cell population was mainly constituted by CD3+/CD8+ cells as well as CD3+/CD56+ NK T cells. Data represent mean ± SEM of 5 different donors (*p* values by *t*-test). **B.** representative flow cytometry dot plots showing transduced CAR T cell sub-populations.

*Ex vivo* stimulated T cells generated clusters with high proliferative capacity that started in the pre-stimulation phase (Fig. 1D, left panel) and was maintained after cell transduction (Fig. 1D, 2 representative donors in the middle and right panels). Gene modified T cells were expanded and further characterized by flow cytometry 15 days after gene transfer. Both GFP only T cells and GD2 CAR T cells were defined by a significant CD3+/CD8+ expansion rate representing the predominant T cell subset, followed by NK T cells expressing both CD3 and CD56. CD3-/CD56+/CD16+ NK cells persisted without significant enrichment throughout the culture (Fig. 2A, 2B).

### GD2 CAR T cells exert specific *in vitro* cytotoxicity against neuroblastoma cells

**Figure 3:**
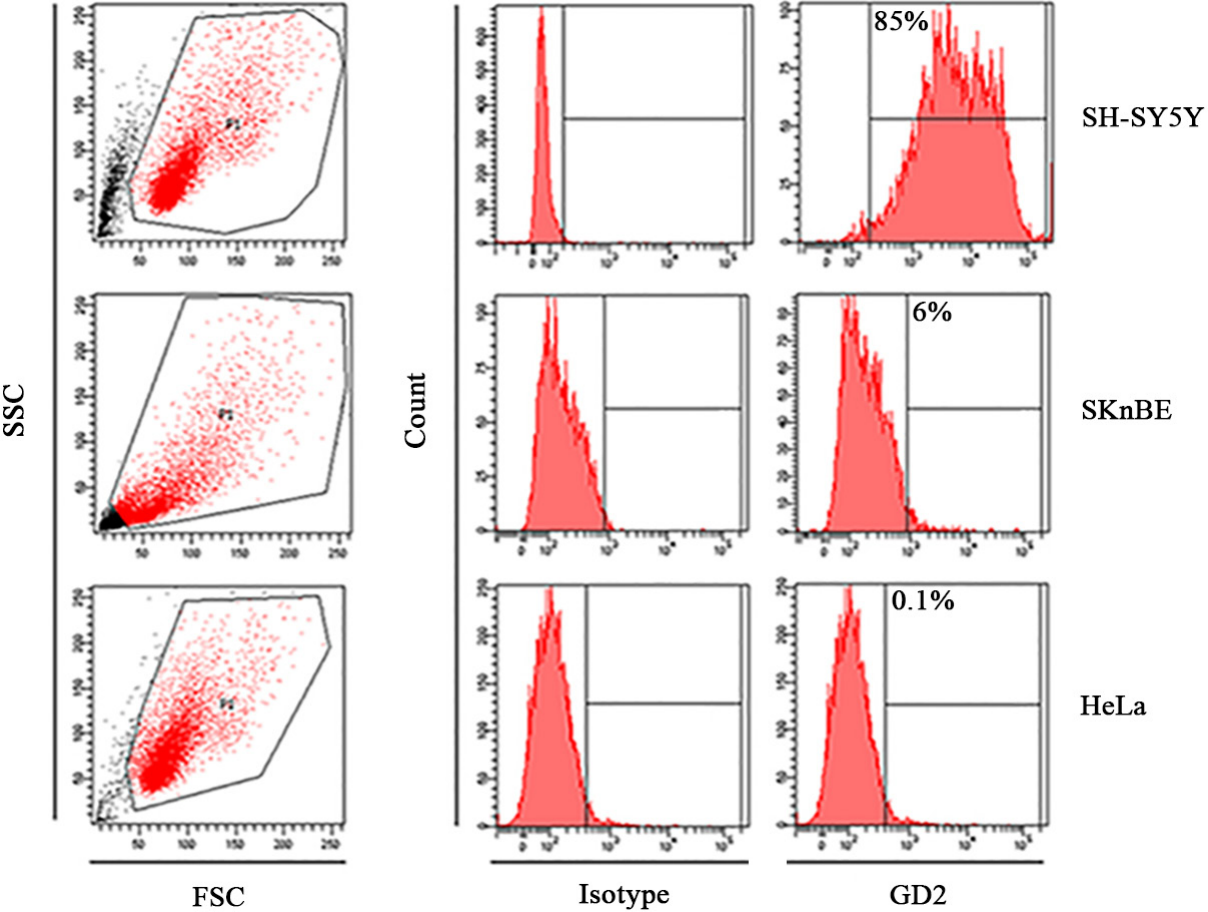
Target cells characterization Representative histograms showing GD2 expression (in red) on human SH-SY-5Y and SKnBE neuroblastoma cell lines and on HeLa cells, the negative control. APC-conjugated secondary Ab was used as isotype.

SH-SY5Y and SKnBE target cell lines were assessed for their GD2 expression in order to be challenged by CAR T cell activity (Fig. 3). High GD2 expression was observed on SH-SY5Y, while low levels were detected on SKnBE. HeLa cell line showed undetectable GD2 levels and was used as negative control.

**Figure 4:**
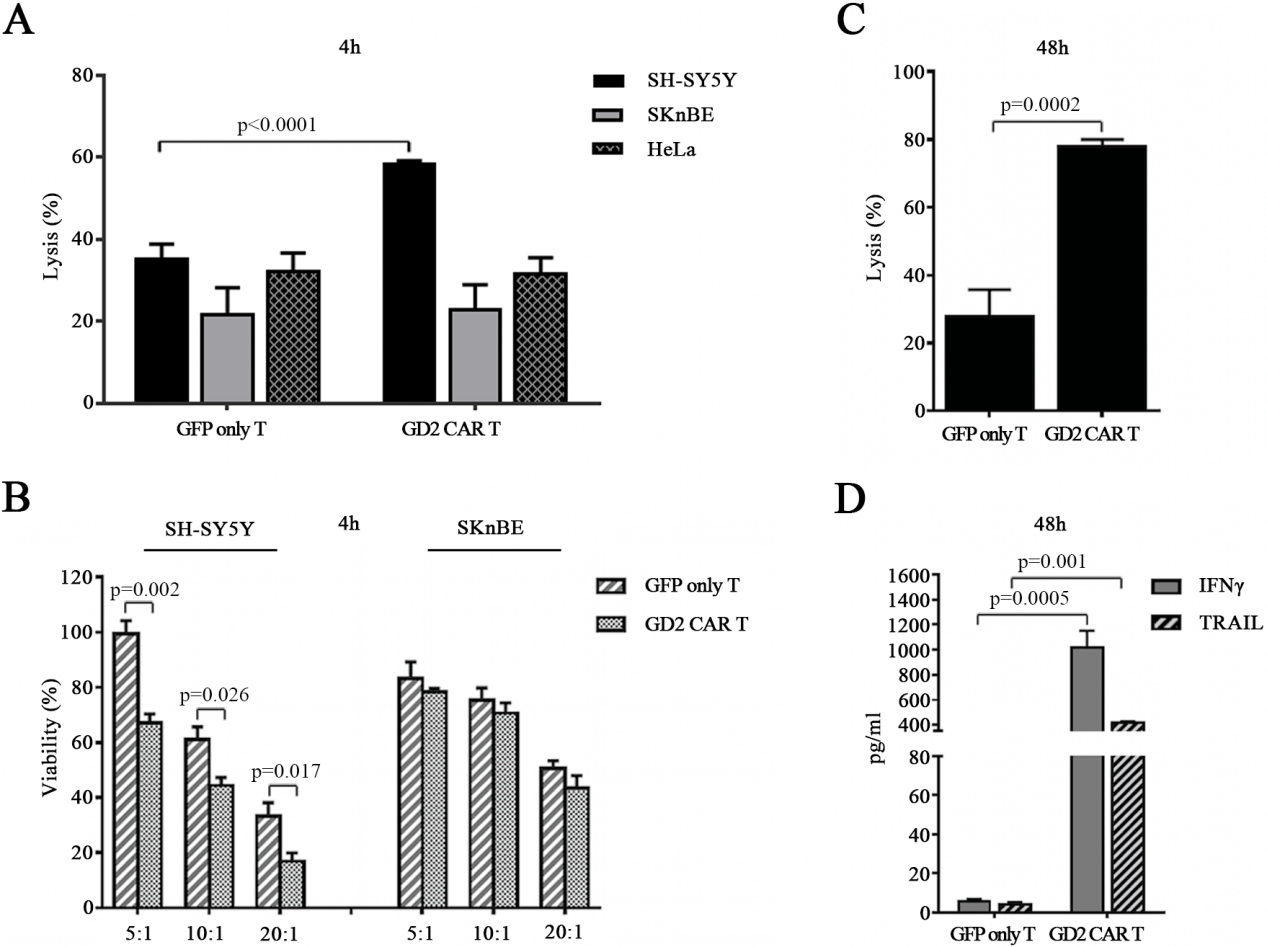
GD2 CAR T cells exert specific *in vitro* cytotoxicity **A.** 4-hour standard ^51^Cr release assay. GFP only T cells and GD2 CAR T cells co-cultured with neuroblastoma cell lines SH-SY5Y, SKnBE or with HeLa cells at E:T ratio of 20:1. **B.** 4-hour CalceinAM viability assay where GFP only T cells and GD2 CAR T cells were co-cultured either with SH-SY5Y or SKnBE cells at E:T ratio of 5:1, 10:1, 20:1. **C.** 48-hour co-culture assay with sorted GD2 CAR T cells (85 ± 5% of purity, not shown) co-cultured with SH-SY5Y at E:T ratio of 1:1. **D.** 48-hour co-culture supernatant concentrations of IFNγ and TRAIL measured by ELISA. All data expressed as mean ± SEM of at least 3 replicates (*p* values by *t*-test).

Once target cells selected, the specific cytotoxicity of unsorted GD2 CAR T cells (transduction efficiency of 48 ± 2% by GFP expression) against neuroblastoma cell lines was first evaluated in a 4-hour ^51^Cr-release assay at E:T ratio of 20:1. GD2 CAR T cells showed significant higher cytotoxicity against SH-SY5Y cells as compared to that exerted by CAR-negative control T cells. There was no substantial difference in cytotoxicity between CAR-positive and CAR-negative T cells when the target cells were the GD2-low or negative cell lines SKnBE and HeLa, respectively (Fig. 4A). Confirming the observed cyotoxicity by ^51^Cr-release, calceinAM-based cytotoxicity assay revealed the specificity of the unsorted GD2 CAR T cells, even at unfavourable conditions such as 5:1 and 10:1. As expected, there was not significant killing against the GD2 low SKnBE cells (Fig. 4B).

To further test the cytotoxic potential of GD2 CAR T cells, SH-SY5Y cells were cocultured for 48 hours with sorted GD2 CAR T cells at a lowest E:T ratio of 1:1 (GD2 CAR T cell selection was performed by sorting GFP-positive cells). After 48 hours of coculture, cells were harvested to be analyzed by FACS and, again, GD2 CAR T cells showed to exert a pronounced cytotoxicity (Fig. 4C). Moreover, the coculture of GD2 CAR T cells with SH-SY5Y cells induced a significant release of both IFNγ and TRAIL by the effector cells (Fig. 4D).

### GD2 CAR T cells provide potent therapeutic activity in xenograft

**Figure 5:**
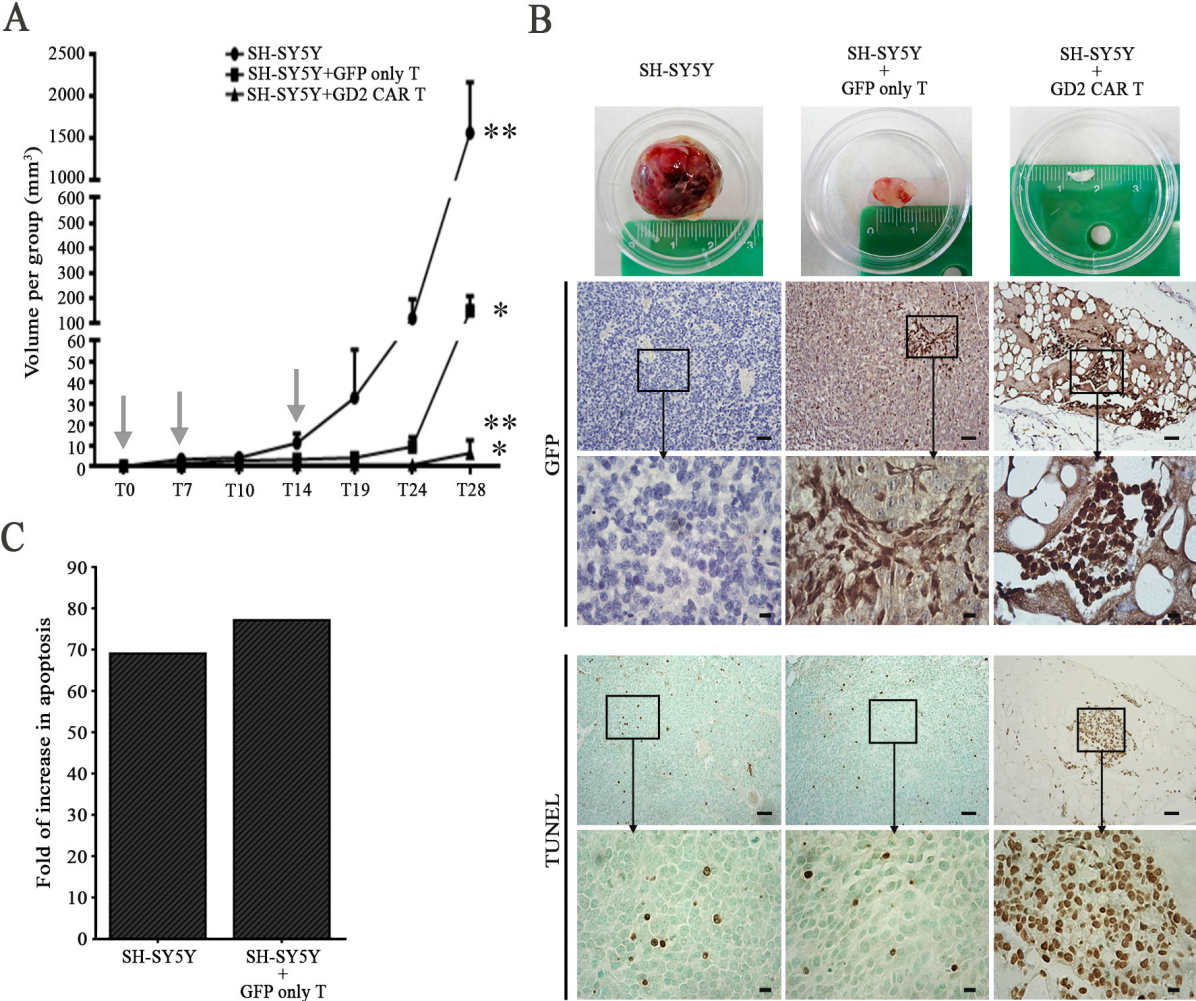
GD2 CAR T cells provide potent therapeutic activity in a neuroblastoma xenograft **A.** GD2 CAR T cells were capable of substantially abrogate tumor growth either versus SH-SY5Y treated with parental GFP only T cells or SH-SY5Y alone (*p = 0.036, **p = 0.027 by *t*-test, mean ± SEM). Arrows represent T cell injection time points. T cells were injected locally into the tumor. **B.** Representative tumors for each group (first row); representative images of anti-GFP staining (second row with insets) of tumor sections obtained from mice injected with SH-SY5Y cells alone (left), SH-SY5Y+GFP only T (middle) or SH-SY5Y+GD2 CAR T cells (right); in the fourth row (with insets) TUNEL assay detecting apoptosis. Scale bars: 50 μm and 10 μm for insets. **C.** Fold of increase in apoptosis of GD2 CAR T cell treated specimen versus either SH-SY5Y alone or SH-SY5Y treated with GFP only T cells.

In the next set of experiments, we assessed GD2 CAR T cell cytotoxicity *in vivo*. SH-SY5Y cells were injected subcutaneously in NOD/SCID mice that were then either injected locally with GD2 CAR T cells or control T cells transduced with GFP only, while another group of mice did not receive T cells. Fig. 5A summarizes sequential tumor volume data. In the untreated group, all mice rapidly developed tumors that reached a mean volume over 1500 mm^3^ in less than 30 days. In mice treated by GFP only T cells, tumor development was observed in all mice but it was overall slower. In contrast, treatment with GD2 CAR T cells suppressed tumor growth in all but one mouse. Harvested tumors (Fig. 5B, first row) were then analysed by hematoxilin & eosin (Supplementary Figure 1) revealing a dramatic change in the histological architecture of specimens treated by GD2 CAR versus controls that displayed large areas containing densely populating neuroblastoma-like cells.

Immunohistochemical analyses of GFP were also performed and, shown in Fig. 5B, clusters of infiltrating GFP positive cells were observed only in tumor sections taken from T cell treated animals (Fig. 5B, second column) and were more prominent in the GD2 CAR treated group (Fig. 5B, third column).

To detect GFP positive circulating T cells in treated mice and controls at the end of the study, DNA from peripheral blood was extracted and a sensitive RT-PCR targeting GFP was performed (Supplementary Figure 2). As expected, no amplification was visualized both for the no template control (NTC) and for SH-SY5Y alone group. In contrast, 60% of samples in the GFP only T cell treated animals displayed faint bands and 80% of samples from the GD2 CAR T cell treated mice revealed a stronger GFP signal suggesting a persistence of modified T cells in the majority of the treated mice.

Finally, TUNEL assay was introduced to assess the degree of apoptosis. Apoptotic cells were sparse in tumor specimens of untreated mice as well as in mice treated with GFP only T cells (Fig. 5B, fourth and fifth row), whereas they were clearly visible in the tumor that developed in the single mouse of the GD2 CAR treated group (Fig. 5B, right column). Image-j analyses confirmed these observations, and GD2 CAR treated group displayed a highly significant increase of apoptosis over SH-SY5Y alone and GFP only T cell treated group (Fig. 5C).

## DISCUSSION

GD2 is overexpressed in neuroblastoma, retinoblastoma, melanoma, small-cell lung cancer, brain tumors, sarcomas and on discrete breast cancer cell sub-populations but it is expressed at low levels on normal tissues, therefore representing a target for immunotherapy [3, 4]. In this context, anti-GD2 immunotherapeutic antibodies have been developed and anti-GD2 CARs have also been reported [13]. We here developed a novel anti-GD2 CAR that, when expressed in T lymphocytes, triggers specific anti-tumor activity against GD2-positive neuroblastoma cells.

Previous studies showed that a small immunoprotein, derived from an IgM (clone 126) and including the anti-GD2 scFv of our CAR, retained affinity and specificity of the original antibody [12]. Therefore, the purpose of this study was to generate a second generation CAR by an anti-GD2 scFv-mouse IgM capable to conjugate the features of anti-GD2 scFv with a potent costimulation driven by 4-1BB molecule. Previously, one IgM and two IgG derived anti-GD2 CARs were tested against neuroblastoma cells either with or without costimulatory signals such as OX40, CD28 and 4-1BB [14, 15, 16]. In our case and for the first time, we selected the anti-GD2 scFv-mouse IgM clone 126 in combination with 4-1BB considering the importance of this costimulatory molecule to sustain cytotoxic T cell activity [17, 18], to favor CD8-positive T cell expansion during either viral infection or allograft rejection [19] and its crucial role in directing the anti-tumor response in animal models and in humans [20]. In particular, an anti-CD19 CAR containing 4-1BB signalling has been shown to produce dramatic responses in patients with B cell malignancies [21].

Our results provided *in vitro* and *in vivo* evidences of a targeted anti–tumor activity toward GD2-positive neuroblastoma cells. Human T lymphocytes were stably transduced by retroviral particles revealing significant levels (exceeding 50% of GFP positivity) at least in line with what was previously reported [16, 22]. The generation of GD2 CAR specific anti-idiotype antibodies allowed us to compare the GFP positivity on transduced T cells with the real CAR surface expression. Taking advantage of this approach, we were able to determine an overlapping signal between GFP and GD2 CAR expression that persisted for at least 30 days (not shown) after transduction. This persistence, when properly translated for clinical applications, may be relevant to rapidly evaluate transduction levels excluding GFP labelling and its related safety concerns after gene modification.

GD2 CAR redirected T cells showed activation, proliferation, and cytokine release upon GD2 stimulation. We firstly introduced the highest E:T ratio into cytotoxic assays in order to identify the most sensitive cell lines. In particular, GD2 CAR T cells exhibited specific targeting versus SH-SY5Y cells as determined by ^51^Cr release in contrast to GD2-negative HeLa cells and against SKnBE neuroblastoma GD2-low expressing cells. Then, the viability of selected target cells, SH-SY5Y and SKnBE, over different E:T ratio was evaluated by CalceinAM 4-hour assay which allows to appreciate the specific targeting versus SH-SY5Y in all culture conditions. We further confirmed the GD2 CAR T cell cytotoxicity versus SH-SY5Y cells over 48-hour coculture assay emphasizing the impact of sorted CAR positive T cells even when a more unfavourable effector to target ratio at 1:1 was tested. While we have observed levels of background in the 4-hour *in vitro* cytotoxicity, the addition of CAR has been constantly associated with a significant increase in killing against SH-SY5Y, the GD2 positive cell line. This high background might be due to the existence of an alloreactivity that may be even enforced by the presence of NK/T (CD3+/CD56+) cells within our effector cell sub-populations. However, in the 48-hour assay, comparing the sorted CAR population with the GFP only T cells, the killing activity background was attenuated and the difference between the two groups was more significantly emerging suggesting the advantage of the CAR within a prolonged allogeneic response, as further confirmed by cytokine release assay and, even more, by the *in vivo* study.

Curiously, after viral transduction we report a proliferation advantage of the NK/T subpopulation in transduced T cells versus non-transduced lymphocytes. The reasons behind this observation would require further investigations. However, it has been reported that the RetroNectin (CH-296), used in the gene modification steps, may enhance a cytokine-induced killer cell expansion in the presence of T cell receptor (TCR)-stimulating signals such as IL-2 or IFNγ [23]. Thus, we can hypothesize that the presence of both CH-296 and IL-2 during transduction could favours the emerging of a NK/T fraction with positive impact on CAR T cell therapeutic profile, as previously described [24].

Anti-cancer killing activity of CAR resides also in cytokines production [16, 25]. We found a significant IFNγ release by GD2 CAR T cells over 48-hour cocultures with SH-SY5Y. In addition, we observed that GD2 CAR T cell action was associated with a dramatic release of a potent death ligand, namely TRAIL. Interestingly, GFP only T cell counterpart did not show these events indicating that CAR expression redirects T cell specificity and reprograms their effector functions against responsive GD2-positive neuroblastoma cells by producing powerful anti-cancer molecules delivered in a combinatory manner.

To evaluate GD2 CAR T cell potential *in vivo*, a subcutaneous neuroblastoma model employing SH-SY5Y cells was adopted. Either GD2 CAR T cells or parental GFP only T cells were administered at the same dose rate to evaluate both antigen specificity and alloreactivity. In mice with SH-SY5Y alone a relevant tumor proliferation took place similarly to what observed in animals treated by allogeneic GFP only T cells. Histology revealed, in the GFP only T cell treated mice, tumor-infiltrating elements suggesting a sub-optimal alloreactivity occurring in mice treated with human T cells. In contrast, GD2 CAR T cells had a powerful anti-tumor activity *in vivo* with a dramatic abrogation of tumor growth in all but one animal.

Others reported animal studies of robust anti-neuroblastoma activity of CAR anti-GD2 human T cells [26, 27], although associated with a xenogenic graft versus host disease (GVHD) in a long-term observation [26]. In our model, animals were sacrificed at 28 days post-injection due to the large size of tumor burden in some of the control mice. While comparisons with these models may be difficult to implement due to a number of variables (i.e. CAR structure, CAR transduction levels, gene engineering methods and *in vivo* study design), we confirmed *in vivo* data without detecting signs of GVHD. Further animal studies shall have to be implemented to further challenge the model, possibly based on *in vivo* imaging. Nevertheless, histological studies of the unique tumor specimens harvested in CAR treated group demonstrate that GD2 CAR T cells were able to robustly infiltrate tumor site inducing apoptosis suggesting the functionality of our approach *in vivo*.

In this *in vivo* study, human T cells were also revealed in mice blood either in GFP only T cell or in GD2 CAR T cell treated animals. While circulating levels of these elements appear low, this aspect is of particular interest indicating that activated T cells can relocate in areas distant from the original intra tumor injection site. In addition, while GFP positive circulating T-cells were present in both groups, only GD2 CAR T cells were able to trigger a significant apoptosis further indicating the relevance of this CAR technology leading to the tumor abrogation through GD2-specific targeting. More in-depth studies are here demanded to better address the dynamic of T cell *in vivo* persistence into blood circulation due to the clinical implications of these findings after transplantation.

With the limitations of a study performed on neuroblastoma cell line only and without comparative studies with other anti-neuroblastoma CAR, our strategy wanted to propose a novel anti-GD2 CAR able to redirect an anti-cancer T cell immune reactivity by-passing tumor escape mechanisms that appear to inhibit the simple alloreactivity. In summary, we have shown that the IgM clone 126-derived GD2 CAR/4-1BB represents a powerful tool to empower T cells towards GD2, supporting future clinical testing of this therapeutic approach in patients with high-risk GD2-positive malignancies.

## MATERIALS AND METHODS

### Cell lines

SH-SY5Y and SKnBE human neuroblastoma cell lines were provided by Pediatric Hospital Gaslini (Genova, Italy) and were maintained in DMEM:F12 (Gibco-Life Technologies, Grand Islands, NY) supplemented with 10% heat-inactivated fetal bovine serum (FBS, Gibco-Life Technologies), 1% L-glutamine (200 mmol/L; Euroclone, Paignton, UK) and 1% penicillin-streptomycin (10000 units penicillin and 10 mg streptomycin/ml in 0.9% sodium chloride, Sigma-Aldrich, Ayrshire, UK). 293T human embryonic kidney fibroblast cell line and FLYRD18 packaging cell line (PCL) were cultured as described [28]. HeLa human cervix adenocarcinoma cell line (kindly provided by Virna Marin, Ospedale S. Gerardo, Monza, Italy), used for viral titration and cytotoxicity studies, was maintained in complete medium composed by DMEM high glucose (Euroclone) with 10% heat-inactivated fetal bovine serum, and 1% penicillin-streptomycin. Tumor cell line authentication was performed by DNA profiling using 8 different and highly polymorphic short tandem repeat (STR) loci (DSMZ-Authentication Service, Braunschweig, Germany).

### Effector cells

Peripheral blood mononuclear cells (PBMC) were separated by density gradient from the peripheral blood of healthy donors after informed consent (Lymphoprep; Fresenius, Axis-Shield, Oslo, Norway) and then plated in RPMI 1640 with 1% FBS, 1% glutamine and 1% penicillin-streptomycin. Non-adherent cells were collected and pre-stimulated for 48 hours in RPMI 1640 supplemented with 10% heat-inactivated defined FBS, 500 UI/mL rhInterleukin-2 (rhIL-2, Proleukin, Novartis Farma S.p.a) and 7 μg/mL Phytohemagglutinin (PHA-M, Sigma-Aldrich) at the concentration of 1 × 10^6^ cells/mL.

### Chimeric antigen receptor construct and vectors

The anti-GD2 mouse heavy (V_H_) and light (V_L_) encoding sequences, derived from the IgM producer hybridoma 126, were previously inserted in the expression vector pcDNA3 generating the pcDNA3-GD2-hεSIP plasmid [12]. The cDNA encoding for the anti-GD2 scFv was then isolated by PCR using gene specific primers: forward 5′-CAGATCTGATGGGCTGGAGCCTGATCCT-3′ and rev 5′TGGCGTCGTGGTAGAGACAGTGACCAG-3′. The signal transduction domain (STD), encoding for the human CD8α hinge-transmembrane domain as well as for a portion of the human 4-1BB molecule and CD3 ζ endodomain, was subcloned from an anti-CD19–41BB-CD3ζ CAR developed at St Jude Children's Research Hospital (Memphis, TN) [17] using the following primers: for 5′-GTCACTGTCTCT ACCACGACGCCAGCGCCG-3′ and rev 5′GGAATTCCTGTGTCTCATAATCTGGGCGTC-3′. The scFv region was subsequently joined to the STD by splicing overlapping extension PCR (SOE-PCR) technique [29] and cloned by TOPO TA Cloning Kit (Invitrogen, Paisley, UK). The whole cDNA encoding for the anti-GD2-BB-ζ CAR was then confirmed by direct sequencing with ABIprism 3100 Genetic Analyzer (Applied Biosystems, Foster City, CA, USA) and the anti-GD2-BB-ζ CAR expression cassette was subcloned into the multiple cloning site of the pMIGR1-IRES-GFP retroviral vector, using BgIII and EcoRI restriction enzymes as described [29] obtaining a pMIGR1-anti-GD2-BB-ζ retroviral vector (GD2 CAR). pMIGR1 vector expressing GFP (GFP only) was used as a control.

### Recombinant retrovirus production and gene transfer

Helper plasmids, pSRαG encoding for the Vesicular Stomatitis Virus (VSV-G) envelope glycoprotein and pMDL encoding for gag-pol genes, were mixed to the respective vector plasmid pMIGR1-anti-GD2-BB-ζ and pMIGR1-IRES-GFP and employed to produce transiently transfected 293T cells as described [28].

To generate stable retrovirus producer cell lines, retroviral supernatants were collected 48 hours after 293T transfection and used to infect the FLYRD18 packaging cell line by overnight incubation at 37°C in the presence of polybrene (6 μg/mL; Sigma-Aldrich). Retroviral supernatants were generated from the FLYRD18 cell line in DMEM supplemented with 10% defined FBS. After 24 hours of incubation at 37°C the supernatants were filtered (0.45 μm PES filter), titrated on HeLa cells and finally employed to transduce pre-stimulated T lymphocytes. Pre-stimulated T cells were transduced by either pMIGR1-anti-GD2–41BB-CD3ζ CAR (“GD2 CAR”) or pMIGR1-IRES-GFP (“GFP only”) retroviral supernatants in the presence of polybrene (4 μg/mL; Sigma-Aldrich) in RetroNectin-coated tubes (rh fibronectin fragment CH-296, Takara Bio, Shiga, Japan) over 6-hour of incubation, for 3 consecutive days. Transduced T cells were then expanded at the concentration of 1 × 10^6^/mL in RPMI 1640 supplemented with 10% heat-inactivated defined FBS, 500 UI/mL rhIL-2 and magnetic beads coated with anti-CD3 and anti-CD28 antibodies (Life Technologies) at 3:1 ratio to T cell.

### Flow cytometry

To assess the immunophenotype, T cells were stained with a panel of dye-conjugated mouse anti-human monoclonal antibodies CD3-PE/APC, CD4-APC, CD8-APC, CD16-PE, CD56-APC and CD45-PE/APC (Becton Dickinson-BD, San Jose, CA). To evaluate GD2 antigen expression, tumor cell lines were stained with a primary unconjugated mouse anti-human Disialoganglioside GD2 (BD) and then with APC-conjugated goat anti-mouse Ig (APC Goat Anti-Mouse Ig polyclonal multiple adsorption; BD). All samples were acquired by BD FACSAria III (BD, Franklin Lakes, NJ), and analysed using BD FACSDiva software. GD2 CAR enriched T cell population was achieved by cell sorting gating on GFP-positive cells.

### Anti-idiotypic antibody production

To detect GD2 CAR expression in T cells, anti-idiotypic antibodies were raised as described [30]. BALB/c mice (*n* = 4) were genetically immunized intradermally by biolistic gold particles coated with the plasmid pcDNA3-GD2-hεSIP coding for the MoAb 126-derived anti-GD2 scFv in the form of a Small Immunoprotein (SIP) [12], using the Gene Gun device (BioRad, Hercules, CA). Three biweekly deliveries for each mouse were performed. Sera from immunized mice were collected 10 days after the last immunization and titrated by flow cytometry for anti-idiotypic antibodies on FLYRD18 packaging cell line expressing GD2 CAR. CAR expression in transduced T cells was tested by anti-idiotype mice sera followed by APC-conjugated goat anti-mouse Ig secondary antibody (BD Pharmingen). T cells were then analysed for both GFP and CAR expression by flow cytometry.

### Cytotoxicity assays

The cytotoxic activity of GD2 CAR and GFP only T cells was tested in a standard 4-hour ^51^Cr release assay as previously described [31]. Isotope release after 4 hour cocultures with effector-to-target (E:T) ratio of 20:1 was assessed by 2450 microplate counter MicroBeta^2^_TM_ (Perkin-Elmer). Target cells (SH-SY5Y, SKnBE and HeLa) were incubated either in complete medium alone or in 1% Triton X-100 (Sigma) to determine the spontaneous and the maximum ^51^Cr release, respectively. Mean percentage of triplicate specific lysis wells was calculated as 100 × (experimental release - spontaneous release)/(maximal release - spontaneous release). Target cell viability was additionally evaluated by a 4-hour calceinAM assay [32]. Target cells (SH-SY5Y and SKnBE) were labeled with calceinAM and cocultured at E:T ratio of 5:1, 10:1 and 20:1. Cultures containing medium alone or 1% Triton X-100 were used as controls, representing 100% and 0% cell viability, respectively. Average viability was calculated as 100 x (experimental fluorescence - 0% viability fluorescence)/(100% viability fluorescence - 0% viability fluorescence). Samples were measured using Victor3 multilabel plate reader (PerkinElmer, Waltham, MA, USA).

The anti-tumor activity of GD2 CAR T cells after GFP sorting was validated in 48-hour assays. SH-SY5Y cells were seeded at E:T ratio of 1:1 with either GFP only T cells or GD2 CAR T cells in RPMI supplemented with 10% FBS, 500 UI/mL rhIL-2 and incubated for 48 hours. Cells were then harvested and stained by both anti-GD2 and anti-CD45 MoAbs specifically identifying target and T cells, respectively. The cytotoxic impact of T cells over 48-hour cocultures was assessed by FACS evaluating persistent CD45-negative/GD2-positive cells and was represented as percentage of lysis.

### ELISA

Interferon gamma (IFNγ) and tumor necrosis factor related apoptosis inducing ligand (TRAIL) levels were measured in supernatants from 48-hour coculture assays, using either Human IFNγ ELISA Kit (Boster immunoleader, Fremont, CA) and Human TRAIL kit assay (R&D System Inc, Minneapolis, MN).

### Xenotransplant models

To assess *in vivo* anti–tumor activity of GD2 CAR T cells, we generated NOD/SCID mouse model (NOD.C.B-17-Prkdc^scid^/J, Charles River Laboratories Italia, SRL, Lecco, Italy) selecting the human SH-SY5Y neuroblastoma cell line as target. Twelve week old mice, six per group, were subcutaneously injected into the flank at day 0 with 1 × 10^6^ SH-SY5Y cells suspended in 0.2 mL Matrigel (BD Biosciences) and at day 0, 7 and 14, either GFP only T cells or GD2 CAR T cells (5 × 10^6^/each time point) were locally injected. Mice were given intraperitoneal injection of 1000 UI/mouse of rhIL-2 (Proleukin, Novartis) once a week. Animal weight and mass growth were monitored till the end of the *in vivo* study at day 28. Longest length and width measurements were recorded and tumor volume was calculated according to the formula (length*width^2^/2). All mouse experiments were done with approval of the local Institutional Animal Care and Use Committee overseeing animal experimentation.

### Histology and TUNEL

Formalin-fixed, paraffin-embedded tumor specimens were cut and stained by hematoxylin-and-eosin staining (Sigma-Aldrich, St Louis, MO). To detect GFP-positive cells, sections were retrieved in citrate buffer (pH 6) for 15 minutes. Primary rabbit polyclonal anti-GFP antibody (1:1200; Abcam: ab290-50) was incubated overnight at 4°C. Slides were then incubated with biotinylated goat anti-rabbit IgG (H+L) (1:200; Vector Laboratories, Burlingame, CA) for 1 hour at room temperature. Negative controls were run simultaneously by omitting primary antibody while incubating with buffer. GFP-positive cells were visualized by diaminobenzidine (DAB, Vector Laboratories). All slides were counterstained with Harris hematoxylin (Bio Optica, Milan, Italy). Sections were examined by Zeiss Axioskop (Zeiss, Oberkochen, Germany). Photomicrographs were acquired by AxioCam ICc3 color camera and AxioVision software (Zeiss). GFP analysis was performed by ImageJ (NIH, Bethesda, MD). TUNEL staining was performed by using Tumor TACS™ *In Situ* Apoptosis Detection Kit (Trevigen, Gaithersburg, MD). Briefly, after deparaffinization and rehydration, specimens were treated with proteinase K solution and quenched with 3% hydrogen peroxide solution. The 3′ ends of cleaved DNA fragments in apoptotic cells are recognized by the terminal deoxynucleotidyl transferase for the incorporation of biotinylated nucleotides. Visualization of chromosomal DNA fragments was allowed by binding of streptavidin-HRP and subsequent DAB incubation. As positive control for apoptosis detection, one section was treated with nuclease to induce DNA fragmentation; TdT enzyme was omitted as a negative control. A nuclear counterstain was performed with methyl green. Sections were examined by Zeiss Axioskop (Zeiss, Oberkochen, Germany). Photomicrographs were acquired by AxioCam ICc3 color camera and AxioVision software (Zeiss).

### Statistics

All *in vitro* and *in vivo* data are expressed by means and SEM. Paired Student's *t* test was used to determine statistical significance. *P* < 0.05 was considered statistically significant.

## SUPPLEMENTARY MATERIALS & METHODS FIGURES AND TABLE


