# Supplementary material for: A novel anti-GD2/4-1BB chimeric antigen receptor triggers neuroblastoma cell killing
Source: Oncotarget. 2015 Jul 20;6(28):24884–94. doi: 10.18632/oncotarget.4670 (PMC4694800; doi:10.18632/oncotarget.4670)
Supplement: Supplementary file 1 [file oncotarget-06-24884-s001.pdf]

SUPPLEMENTARY MATERIALS & METHODS

RT-PCR

To detect the presence of circulating human modified T cells, GFP signal was tracked. At the end of the *in vivo* study (on day 28), 500 µl of blood were collected and genomic DNA was purified by MasterPure™ DNA Purification Kit for Blood Version II (Epicentre, Illumina, Madison, WI). DNA from cultured GFP only T cells and GD2 CAR T cells was isolated using QIAamp DNA Mini purification kit (Qiagen, Hilden, Germany). All samples were amplified to detect both the mGAPDH, as housekeeping for murine DNA, and the GFP sequence encoded by the retroviral vector used to transduce *T* cells. Primers (IDT software, Leuven, Belgium; Clone Manager, Informer Technologies Inc, Morrisville, NC) are listed in Supplementary Table S1. Twenty nanograms of template were amplified using fast SYBR® green master mix (Applied Bio-systems, Thermo Fisher Scientific, Waltham, MA) performed by Step OneTM Real Time PCR System (Applied Biosystems, Thermo Fisher Scientific, Waltham, MA) at the following conditions: 20'' at 95°C followed by 40 amplification cycles (95°C for 3'' and 60°C for 30''). Final melt curve stage has been performed. The no-template control (NTC) consisted of all PCR reaction mixture except template. GFP negative control was obtained from SH-SY5Y alone group, while GFP positive control was isolated from cultured GFP only T cells and GD2 CAR T cells. All measurements were performed twice. Analyses were assessed by Step OneTM software and by electrophoresis through 1.8% (wt/vol) agarose gels.

SUPPLEMENTARY FIGURES AND TABLE

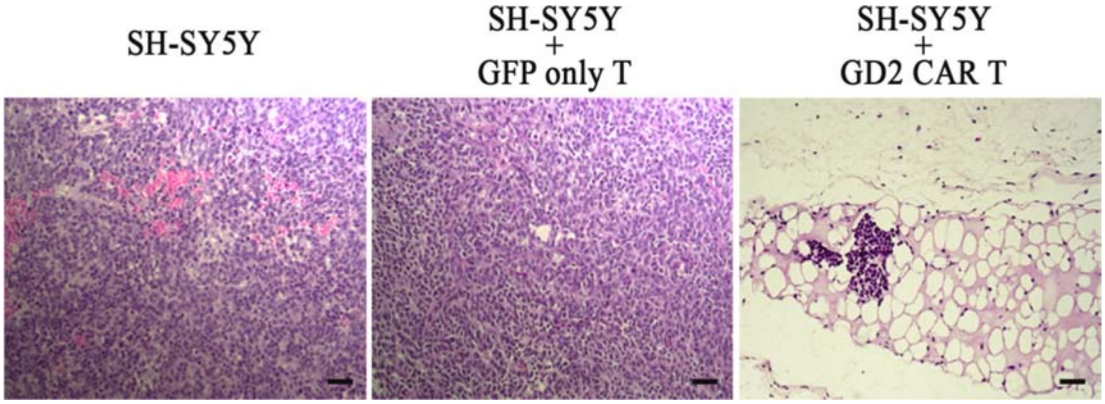

Supplementary Figure S1: Harvested tumors were analysed by hematoxilin & eosin revealing a dramatic change in the histological architecture of specimens treated by GD2 CAR versus SH-SY5Y alone and GFP only T cell treated groups that displayed large areas containing densely populating neuroblastoma cells. Scale bars: 50 µm.

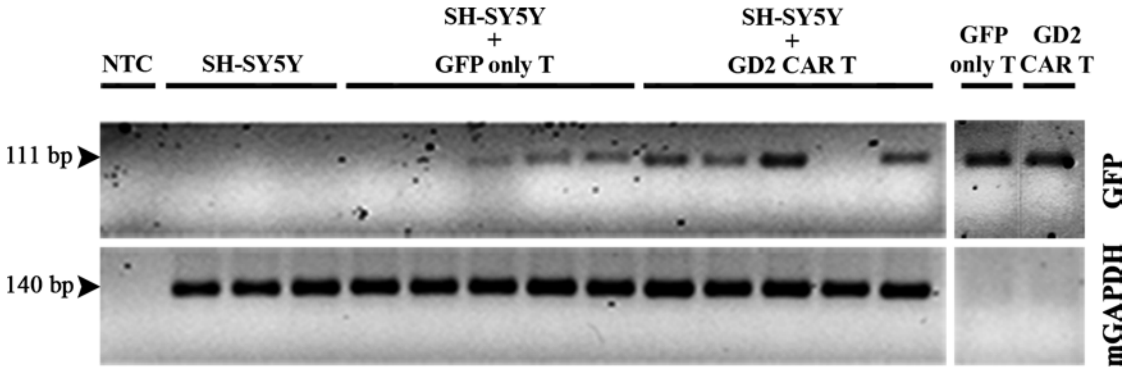

Supplementary Figure S2: Circulating effector cells persist within the blood of transplanted mice. Agarose gels of GFP and murine GAPDH (mGAPDH) amplificated DNA fragments. Sample lanes are as follow: lane 1, no-template control (NTC); lanes 2 to 4, SH-SY5Y alone group; lanes 5 to 9 GFP only T cell treated group; lanes 10 to 14 GD2 CAR T cell treated group, lanes 15 and 16, cultured GFP only T cells and GD2 CAR T cells, respectively. Band size (bp) of amplified DNA fragments on the left.

Supplementary Table S1: Primers used in the RT-PCR

Gene	Primer sequence	Expected amplified length
**GFP**	5′-GAA CCG CAT CGA GCT GAA-3′ (sense)<br>5′-TGC TTGTCG GCC ATG ATA TAG-3′ (antisense)	111 bp
**mGAPDH**	5′-GCC TTC CGT GTT CCT ACC-3′ (sense)<br>5′-CCT CAG TGT AGC CCA AGA TG-3′ (antisense)	140 bp
